# Supplementary material for: Knowledge and preventive practices towards COVID-19 among pregnant women seeking antenatal services in Northern Ghana
Source: PLoS One. 2021 Jun 17;16(6):e0253446. doi: 10.1371/journal.pone.0253446 (PMC8211189; doi:10.1371/journal.pone.0253446)
Supplement: S1 Appendix — (DOCX) [file pone.0253446.s001.docx]

**S1 Appendix. Questions on knowledge of COVID-19**

|  | **Variables** | **Yes** | **No** |
| --- | --- | --- | --- |
| 1 | Can COVID-19 spread from person-to-person within close distance of each other (less than one meter)? |  |  |
| 2 | Can COVID-19 spread through respiratory droplets, which occur when infected people cough and sneeze? |  |  |
| 3 | Can COVID-19 can be contracted by touching a surface or object, on which the virus is attached, and then touching one’s mouth, nose, or, perhaps, eyes? |  |  |
| 4 | Can COVID-19 present with symptoms such as fever, tiredness, dry cough and shortness of breath |  |  |
| 5 | Do you know that unlike the common cold, congestion, runny nose, and sneezing are less common in people infected with COVID-19? |  |  |
| 6 | Do you know that pregnant women are more susceptible to infections than non-pregnant women? |  |  |
| 7 | Do you know that after visiting a public place, after nose-blowing, coughing or sneezing, people must wash their hands with soap and water, or use alcohol-based hand sanitizer? |  |  |
| 8 | Do you know that people should avoid touching their eyes, nose, and mouth with unwashed hands to prevent COVID-19? |  |  |
| 9 | Do you know that wearing of facemask can prevent COVID-19 infection? |  |  |
| 10 | Do you know that avoiding crowded places can prevent transmission of COVID-19 |  |  |
