# Supplementary material for: Knowledge and preventive practices towards COVID-19 among pregnant women seeking antenatal services in Northern Ghana
Source: PLoS One. 2021 Jun 17;16(6):e0253446. doi: 10.1371/journal.pone.0253446 (PMC8211189; doi:10.1371/journal.pone.0253446)
Supplement: S2 Appendix — (DOCX) [file pone.0253446.s002.docx]

**S2 Appendix. Questions on COVID-19 preventive practices**

1. Do you use facemask in public places?

1. Yes [ ]
2. No [ ]

2. Do you practice hand washing with water and soap or alcohol-base sanitizer because you want to prevent COVID-19?

1. Yes [ ]
2. No [ ]

3. Do you observe physical distancing (at least 1 meter) when in public space?

1. Yes [ ]
2. No [ ]

4. Do you cough or sneeze into a tissue or elbow when in public?

1. Yes [ ]
2. No [ ]

5. Do you avoid touching your eyes, nose and mouth with unwashed hands?

1. Yes [ ]
2. No [ ]
