# Supplementary material for: Knowledge and preventive practices towards COVID-19 among pregnant women seeking antenatal services in Northern Ghana
Source: PLoS One. 2021 Jun 17;16(6):e0253446. doi: 10.1371/journal.pone.0253446 (PMC8211189; doi:10.1371/journal.pone.0253446)
Supplement: Attachment — Submitted filename: Response to Reviewers.docx [file pone.0253446.s004.docx]

**Reviewer #1**

COVID-19 is a novel respiratory disease associated with severe morbidity and high mortality in the elderly population and people with comorbidities. Studies have suggested that pregnant women are more susceptible to COVID-19 compared to non-pregnant women. However, it’s unclear whether pregnant women in Ghana are knowledgeable about COVID-19 and practice preventive measures against it.
This manuscript I provides evidence of knowledge of COVID -19 among pregnant women in Ghana. The Manuscript is technically sound, well written and data analysis supports the conclusion.

**Response:** We sincerely appreciate the reviews.

**Reviewer #2**

The study was carried out in Ghana in the Nabdam district in the Northern part of the country. It sought to find out whether pregnant women we putting themselves at risk of COVID-19 in terms of following the stipulated COVID-19 preventive measures and also to find out whether they are in the known at all of these preventive measures. Questionnaires were administered as the pregnant women visited the hospital for antenatal care.

**Response**

- We sincerely appreciate the reviews.

The northern part of Ghana in terms of demography and topology is totally different from the southern part so the title is misleading.

**Response**

- We have changed the title to reflect Northern Ghana. See lines 1 and 2.

What informed in the selection of the particular health facilities?

**Response**

- All health facilities in the district that conducts antenatal care services were selected for the study. See lines 113 and 114.

How many health facilities were selected?

**Response**

- 16 health facilities were selected. See lines 113 and 114.

How dispersed were they?

**Response**

- The health facilities were not so dispersed. The distance between health facilities ranged from three to seven kilometers.

How can this be a representative study for the whole district. The scope of sampling is too small to generalize to the whole district.

**Response**

- Although the health facilities were purposively sampled, they included all antenatal care facilities in the district. Furthermore, pregnant women were randomly sampled from all these health facilities in the district. We therefore think that our findings can generalized in the district although same may not be applied in the country. We have stated it in our limitations. See lines 255, 256 and 257.

How is the attitude of the pregnant women in terms of attending antenatal?

**Response**

- Our study did not assessed attitude of pregnant women towards antenatal care attendance. However, some of the authors are practitioners in the district and have observed that antenatal care attendance is high.

The authors per the last paragraph states that no studies on knowledge and preventive practices are focused on pregnant women, but this is not so. So many studies on knowledge and preventive practices focused on pregnant women have been carried out by several people Nwafor et al 2020, Anikwe et al 2020, Fikadu et al 2021, Kassie et al 2021 among others. Authors should therefore state in clearer terms the novelty of the study?

**Response**

- This study is the first of its kind in Ghana. We have appropriately stated that in the paper. See lines 97 and 98.

Authors should check grammar, there are a lot of grammatical errors in the article.

**Response**

- We have thoroughly read the paper and grammatical errors have been corrected.

Authors should number the lines of the article to make it easier to make reference to.

**Response**

- We have numbered all the lines of the article.

The conclusion at the abstract section was just a result restated. Authors should conclude appropriately.

**Response**

- The conclusion has been reconstructed appropriately. See lines 43,44 and 45.
