# Supplementary material for: Knowledge and preventive practices towards COVID-19 among pregnant women seeking antenatal services in Northern Ghana
Source: PLoS One. 2021 Jun 17;16(6):e0253446. doi: 10.1371/journal.pone.0253446 (PMC8211189; doi:10.1371/journal.pone.0253446)
Supplement: Attachment — Submitted filename: Response to Reviewer.docx [file pone.0253446.s005.docx]

Reviewer #1: (No Response)

**Response:**

We sincerely appreciate your reviews.

Reviewer #2: For the conclusion, state the type of education on COVID-19 needed to be given pregnant women that is not being done already.

**Response:** Education of COVID-19 preventive practices at the health facility level has been emphasized. See page 3 line 44.

Abstract should emphasize on the Northern Ghana.

**Response:**

This has been edited. See page 2 line 27

At the onset of the study there might not have been COVID-19 vaccines. Now there are, authors should kindly update this information in their introduction.

**Response:**

This information has been updated. See page 5 lines 84-86 and lines 91-92.

At the discussion section COVID-19 was mistakenly written as COVID-1, edit.

**Response:**

This has been edited. See page 12 line 204

How does having at least primary education associate with easier access to health information compared to individuals without a formal education, explain further.

**Response:**

It exposes mothers to knowledge on health compared to mothers with no formal education. We have cited this sentence in page 13 line 221.
