# Supplementary material for: Knowledge and preventive practices towards COVID-19 among pregnant women seeking antenatal services in Northern Ghana
Source: PLoS One. 2021 Jun 17;16(6):e0253446. doi: 10.1371/journal.pone.0253446 (PMC8211189; doi:10.1371/journal.pone.0253446)
Supplement: Attachment — Submitted filename: Response to reviewer.docx [file pone.0253446.s006.docx]

Reviewer #3:

This study aimed to assess the knowledge and preventive practices towards COVID-19 among pregnant women seeking antenatal services in Northern Ghana. A cross-sectional study was conducted using a structured questionnaire in the Nabdam district in Ghana. A total of 527 pregnant women were randomly sampled from health facilities offering antenatal care services in the district. Multivariable logistic regression analysis was used to assess the association between the predictors and outcome variables. Although the majority of the women had adequate knowledge of COVID-19, less than half of them were engaged in good COVID-19 preventive practices. Education of pregnant women on COVID-19 preventive practices should be intensified at health facilities while improving upon the water, sanitation and hygiene need particularly in rural communities.
This study has merit and important in the context of Ghana. However, I have several comments on the content.

**Response:**

We sincerely appreciate your reviews.

**Major Comments:**
1. “The sample size for the study was estimated using EpiInfo Version 7.1 (STAT CALC). The prevalence of knowledge on COVID-19 was not known, therefore a 50% prevalence rate was used with a 95% confidence interval and 5% margin of error. The estimated minimum sample size was 407 including a 10% non-response rate. However, 527 participants completed our surveys.” Why was there oversampling and how this might affect the study?

**Response:**

The sample size of 407 was the minimum sample size required. A sample size of 527 will increase the precision of our estimates.

2. Line 139-140: “A score of 3 points and above was considered good COVID-19 preventive practices while 2 points and below was considered poor COVID-19 preventive practices.” Was this cut-off validated? Please provide reference of previous literature who used this score.

**Response:**

This cut-off was adapted from Bloom’s cut-off points. It was duly cited in our study. See page 7 line 139.

**Minor Comments:**
1. There are some typos in the manuscript. For example, in line 90 “Ghana reported its first case of COVIG-19 on 12th March 2020 in its national capital, Accra [21].” Please change this to COVID-19.

**Response:** This error has been corrected. See page 5 line 90. We have also read through the manuscript and made all the necessary correction of errors.

2. Line 121: add the unit (years) after the age.

**Response:**

Years has added accordingly. See page 6 lines 121-122.
